# Development of Augmented Reality Vision for Osteosynthesis Using a 3D Camera

**DOI:** 10.7759/cureus.60479

**Published:** 2024-05-17

**Authors:** Junichiro Morita, Akira Ikumi, Takushi Nakatani, Hiroshi Noguchi, Hajime Mishima, Tomoo Ishii, Yuichi Yoshii

**Affiliations:** 1 Graduate School of Medicine, University of Tsukuba, Tsukuba, JPN; 2 Department of Orthopaedic Surgery, University of Tsukuba, Tsukuba, JPN; 3 Department of Orthopaedic Surgery, Showa General Hospital, Kodaira, JPN; 4 Department of Orthopaedic Surgery, Tokyo Medical University Ibaraki Medical Center, Ami, JPN

**Keywords:** osteosynthesis, preoperative plan, computed tomography, 3d camera, augmented reality (ar)

## Abstract

Background: We developed a 3D camera system to track motion in a surgical field. This system has the potential to introduce augmented reality (AR) systems non-invasively, eliminating the need for the invasive AR markers conventionally required. The present study was performed to verify the real-time tracking accuracy of this system, assess the feasibility of integrating this system into the surgical workflow, and establish its potential to enhance the accuracy and efficiency of orthopedic procedures.

Methods: To evaluate the accuracy of AR technology using a 3D camera, a forearm bone model was created. The forearm model was depicted using a 3D camera, and its accuracy was verified in terms of the positional relationship with a 3D bone model created from previously imaged CT data. Images of the surgical field (capturing the actual forearm) were taken and saved in nine poses by rotating the forearm from pronation to supination. The alignment of the reference points was computed at the three points of CT versus the three points of the 3D camera, yielding a 3D rotation matrix representing the positional relationship. In the original system, a stereo vision-based 3D camera, with a depth image resolution of 1280×720 pixels, 30 frames per second, and a lens field of view of 64 specifications, with a baseline of 3 cm, capable of optimally acquiring real-time 3D data at a distance of 40-60 cm from the subject was used. In the modified system, the following modifications were made to improve tracking performance: (1) color filter processing was changed from HSV to RGB, (2) positional detection accuracy was modified with supporting marker sizes of 8 mm in diameter, and (3) the detection of marker positions was stabilized by calculating the marker position for each frame. Tracking accuracy was examined with the original system and modified system for the following parameters: differences in the rotation matrix, maximum and minimum inter-reference point errors between CT-based and camera-based 3D data, and the average error for the three reference points.

Results: In the original system, the average difference in rotation matrices was 5.51±2.68 mm. Average minimum and maximum errors were 1.10±0.61 and 15.53±12.51 mm, respectively. The average error of reference points was 6.26±4.49 mm. In the modified system, the average difference in rotation matrices was 4.22±1.73 mm. Average minimum and maximum errors were 0.79±0.49 and 1.94±0.87 mm, respectively. The average error of reference points was 1.41±0.58 mm.

In the original system, once tracking failed, it was difficult to recover tracking accuracy. This resulted in a large maximum error in supination positions. These issues were resolved by the modified system. Significant improvements were achieved in maximum errors and average errors using the modified system (P<0.05).

Conclusion: AR technology using a 3D camera was developed. This system allows direct comparisons of 3D data from preoperative CT scans with 3D data acquired from the surgical field using a 3D camera. This method has the advantage of introducing AR into the surgical field without invasive markers.

## Introduction

Augmented reality (AR) is a technology that seamlessly superimposes 3D image data onto the real world. This technology allows the superimposition of graphics, including text, medical imaging, and preoperative templating, into the surgeon's visual field in 3D and real time. It is now being implemented in the field of orthopedic surgery [1]. In joint replacement surgery, the AR visualization of anatomical structures has led to improved accuracy in the positioning of implants [2-7]. In spine surgery, AR navigation systems have been developed to assist in the precise insertion of intervertebral devices [8-12]. In trauma care, increases in the placement accuracy of implants and the decreased use of X-rays with AR, mainly in the treatment of pelvic fractures, have been reported [13-15]. These applications aim to enable surgeons to perform more precise operations, ultimately improving clinical outcomes. Despite these advances, the widespread adoption of AR technology is still in its infancy, and its utility and effectiveness continue to be evaluated.

There are still several challenges associated with introducing AR technology into orthopedic surgery. First, a significant hurdle is the static nature of preoperative planning data, which does not consider dynamic changes during actual surgery [16]. Second, there are potential issues with movement in the targeted surgical field or obstructions to the surgeon's view during procedures [17,18]. Third, the accuracy and reliability of AR systems often lack sufficient clinical validation. Technological improvements and comprehensive clinical trials are required to address these issues. Finally, to implement AR technology in practical use, user comfort, data updates in response to changes in the field of view, and smooth integration with visual devices are necessary.

3D camera technology was recently introduced to obtain not only vertical and horizontal information but also depth information [19,20]. A 3D camera captures and analyzes 3D information on the surgical field, allowing alignment with the preoperative simulation model. It enables the instantaneous adaptation of preoperative anatomical data to the changing clinical situation during surgery. Therefore, real-time tracking with 3D cameras is one of the solutions currently being applied to AR technology.

We developed a 3D camera system to track motion in a surgical field. This system tracks movement in a surgical field by comparing 3D data based on the surgical field with 3D data from previous CT scans. This system has the potential to introduce AR systems non-invasively, eliminating the need for the invasive AR markers conventionally required. The present study was performed to validate the real-time tracking accuracy of the forearm model using the developed system, assess the feasibility of integrating this system into the surgical workflow, and establish its potential to enhance the accuracy and efficiency of orthopedic procedures.

## Materials and methods

CT imaging and creation of 3D data of a forearm model

**Figure 1:**
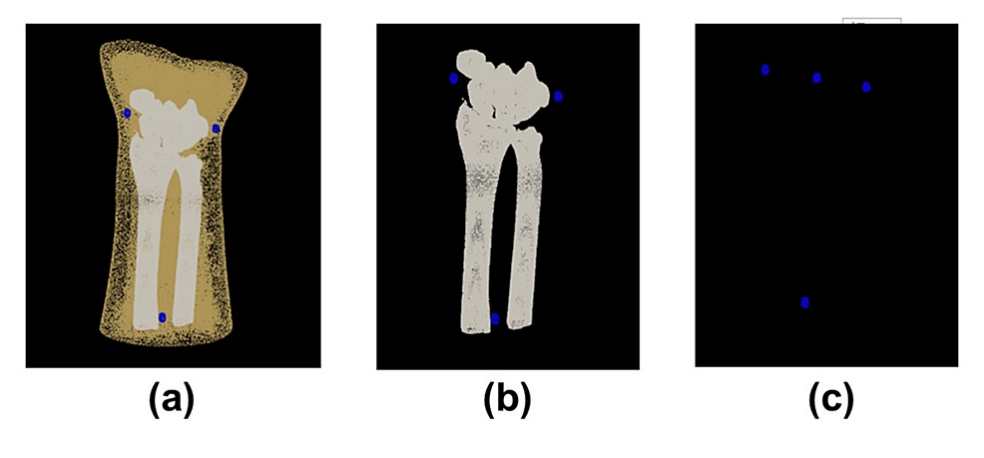
3D data for AR 3D bone images of the forearm model consisting of forearm STL, including the epidermis (a), bone STL (b), and marker STL (c). AR: augmented reality; STL: stereolithography

To evaluate the accuracy of AR technology using a 3D camera, a bone model simulating the forearm was created. The present study was exempt from IRB approval because it was an experimental study using a bone model. A normal forearm model was depicted using a 3D camera, and its accuracy was verified in terms of the positional relationship with a 3D bone model created from previously imaged CT data. The bone model was made of epoxy resin and was created with surface processing to be depicted by X-rays (TANAC Co., Ltd.). The bone model was covered with an X-ray transparent elastic material (urethane resin) simulating skin. Stickers for identifying reference points on the body surface by color and metal ball markers on their surfaces were placed on the forearm model, and a CT scan was performed. CT images were taken at 120 kV, 100 mAS, a slice thickness of 0.8 mm, and a pixel size of 0.3×0.3 mm (Sensation Cardiac, Siemens). 3D bone images of the forearm model were created from the Digital Imaging and Communications In Medicine dataset of the CT scan using image analysis software (ZedView, LEXI Co., Ltd.). 3D bone images of the forearm model consisted of forearm stereolithography (STL), including the epidermis, bone STL, and marker STL (Figure 1).

**Figure 2:**
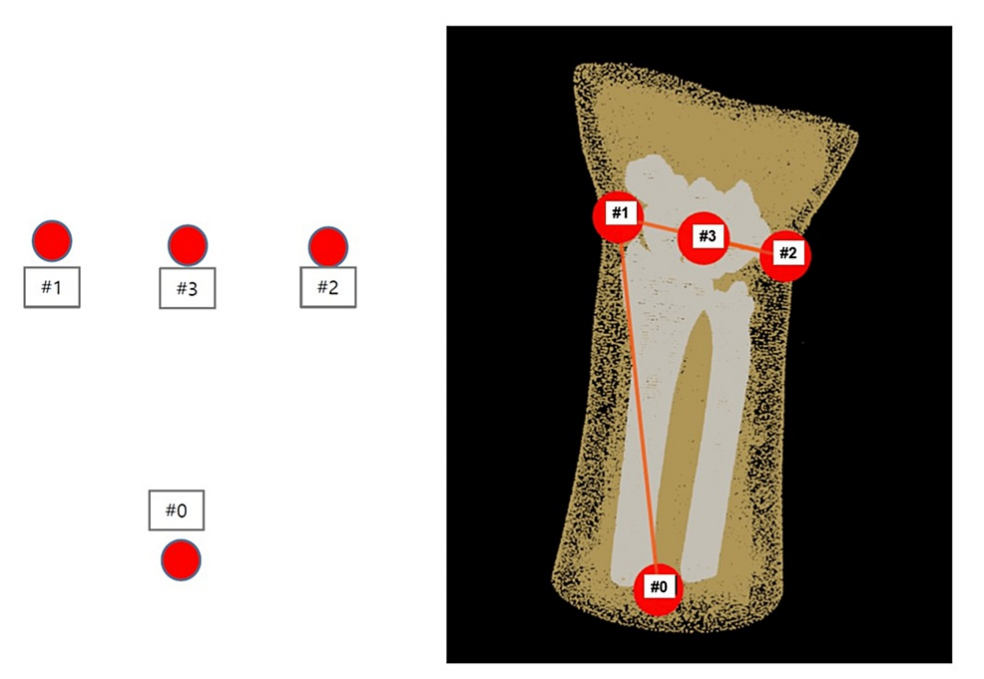
Marker placement In the present study, we adopted a method of attaching four markers to the forearm, consisting of three points around the wrist (markers #1, #2, and #3) and one point at the lower center (marker #0).

The camera used was a stereo vision-based 3D camera, with a depth image resolution of 1280×720 pixels, 30 frames per second, and a lens field of view (FOV) of 64 specifications, with a baseline of 3 cm, capable of optimally acquiring real-time 3D data at a distance of 40-60 cm from the subject (aeroTAP, nextEDGE Technology). In the present study, four reference markers were applied to the surface of the forearm: three around the wrist (markers #1, #2, and #3) and one at the bottom center (marker #0), as shown in Figure 2. This assumes that the lower marker #0 is always captured by the camera, even if the forearm rotates, ensuring that at least three points including the lower marker #0 are captured by the camera. These three points are used to align the preoperative forearm 3D model (in computer space) with the real forearm 3D model constructed in real time by the 3D camera (in real space).

During the alignment, a 4×4 rotation matrix representing the spatial relationship between the spaces is obtained through singular value decomposition, selecting the closest result from the distances of each pair of results, based on the combination of three points that are formed from the four markers in the preoperative forearm space, and three or four markers observable in the real forearm 3D space generated by the 3D camera. If the base marker #0 on the real forearm 3D model cannot be observed or if the number of observable markers is less than three, the result is considered indeterminate.

**Figure 3:**
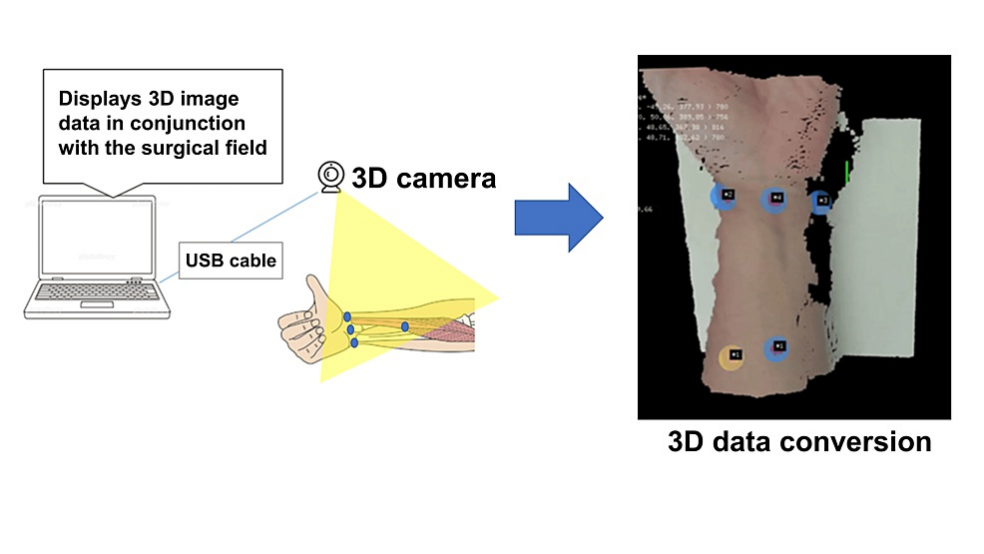
3D camera setting and data conversion The 3D camera is positioned facing the forearm from the front. Markers are extracted from the actual forearm 3D model using marker colors (blue markers on the model surface). The positional relationship between the markers of the preoperative forearm 3D model and the actual forearm 3D model captured by the 3D camera is calculated.

As shown in Figure 3, the 3D camera is positioned facing the forearm from the front, including markers attached to the forearm and epidermis, to digitize the actual forearm in real time as 3D data. The markers are extracted from the actual forearm 3D model using marker colors (refer to Figure 3 for the 3D data of the actual forearm generated by the 3D camera). The positional relationship between the markers of the preoperative forearm 3D model and the actual forearm 3D model captured by the 3D camera is calculated. By processing the alignment calculations frame by frame, real-time tracking becomes possible.

**Figure 4:**
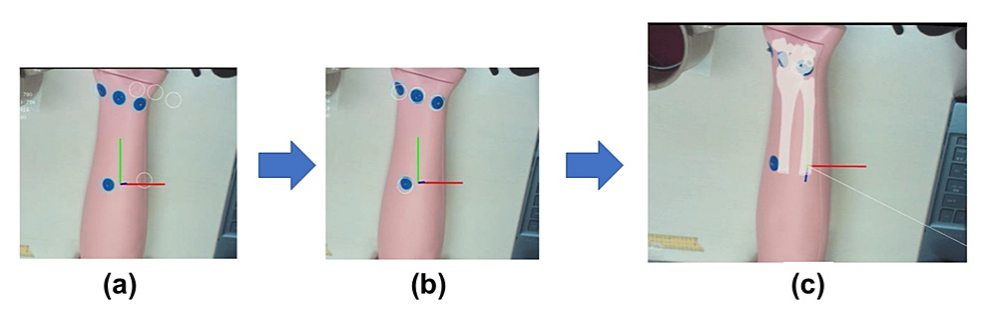
Process of AR visualization (a) Before the alignment, (b) after the alignment, and (c) 3D data were displayed after the alignment. AR: augmented reality

Once the positional relationship between the computer space and the real space is clarified, it is possible to overlay preoperative 3D on the actual forearm 3D, allowing the overlaid 3D model to be displayed in AR. The ultimate goal of this project is to overlay the preoperative forearm model on the surgical field using AR glasses; however, at the current stage, the field of view of the 3D camera has been output as the surgical field. Markers may similarly be used to align the actual forearm 3D model with the surgical field. By calculating the positional relationship between the surgical field and the 3D image and projecting the 3D image onto the surgical field, it was possible to overlay the preoperative forearm 3D image in real time on the surgical field (Figure 4). The preoperative forearm image being overlaid rotated in accordance with the rotation of the actual forearm.

During the process of development, the system was modified to improve tracking accuracy. Improvements were achieved by enhancing the processing speed and obtained high-resolution 3D depth information. At the same time, the 3D camera was adapted to a modified version with an image sensor size of 1/3 inch and a lens FOV of 50, with a baseline of 6 cm. To improve tracking performance, the following modifications were performed: (1) color filter processing was changed from HSV to RGB, (2) positional detection accuracy was modified with supporting marker sizes of 8 mm in diameter (the original system had a support marker size of 20 mm), and (3) the detection of marker positions was stabilized by calculating the marker position for each frame, tracking it, and identifying the position through a moving average.

**Figure 5:**
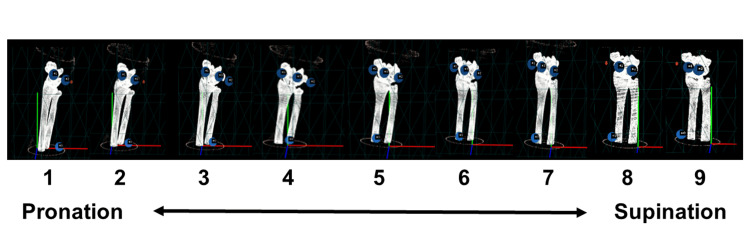
Forearm positions for the analysis Images of the forearm model were taken and saved in nine poses by rotating the forearm from pronation to supination by approximately 10 degrees each.

The alignment error between the computer space and real space was measured using the following method. In this system, the forearm was rotated from pronation to supination, and images of nine poses in the surgical field were captured and stored at approximately 10 degrees each (Figure 5). The alignment of the reference points was computed at the three points of CT versus the three points of the 3D camera, yielding a 3D rotation matrix representing the positional relationship. The error between the marker positions obtained from the rotation matrices calculated for these image alignments and the actual positions was evaluated using the following parameters: (1) difference in the rotation matrix (mm), (2) maximum and minimum inter-reference point errors between CT-based and camera-based 3D data (mm), and (3) average error for the three reference points (mm).

Statistical analysis

The accuracies of position estimations were compared between the original system and the modified system. Results are expressed as the mean±standard deviation. The Shapiro-Wilk test was used to test the normality of datasets. Parameters were compared between the original system and the modified system using Welch's t-test. P-values <0.05 were considered to be significant. All statistical analyses were performed using BellCurve for Excel, Version 2.12 (SSRI Co., Tokyo, Japan), and IBM SPSS Statistics for Windows, Version 24.0 (Released 2016; IBM Corp., Armonk, New York, United States).

## Results

The results obtained on tracking accuracy for each position are shown in Table 1. In the original system, the average difference in rotation matrices was 5.51±2.68 mm. Average minimum and maximum errors were 1.10±0.61 and 15.53±12.51 mm, respectively. The average error of reference points was 6.26±4.49 mm.

**Table 1:** Results of tracking accuracy for each position *: significant differences between the original system and the modified system (P<0.05)

		Differences of rotation matrices (mm)		Min/max errors of reference points (mm)		Average errors of reference points (mm)	
	Positions	Original	Modified	Original	Modified	Original	Modified
Pronation	Position 1	2.84	3.42	0.49/24.36	0.46/1.59	8.65	1.14
	Position 2	4.20	2.46	1.07/2.04	0.39/1.13	1.40	0.82
	Position 3	3.97	2.91	0.96/1.97	0.59/1.27	1.32	0.97
	Position 4	6.74	3.46	0.91/3.26	0.68/1.45	2.25	1.15
	Position 5	5.00	3.67	0.62/2.42	0.44/1.67	1.67	1.22
	Position 6	1.62	2.85	0.34/23.73	0.50/1.34	8.19	0.95
	Position 7	6.86	5.61	1.81/26.52	0.84/2.42	10.45	1.87
	Position 8	8.90	6.17	2.05/27.44	1.72/2.92	11.11	2.06
Supination	Position 9	9.50	7.40	1.66/28.04	1.53/3.67	11.30	2.47
	Ave	5.51	4.22	1.10/15.53*	0.79/1.94*	6.26*	1.41*
	SD	2.68	1.73	0.61/12.51	0.49/0.87	4.49	0.58

In the modified system, the average difference in rotation matrices was 4.22±1.73 mm. Average minimum and maximum errors were 0.79±0.49 and 1.94±0.87 mm, respectively. The average error of reference points was 1.41±0.58 mm.

In the original system, once tracking failed, it was difficult to recover tracking accuracy. This resulted in a large maximum error in supination positions. This issue was resolved by the modified system. Significant improvements were achieved in maximum errors and average errors using the modified system (P<0.05).

## Discussion

The application of AR in the medical field spans from surgical planning to education and rehabilitation; however, its implementation and development face several challenges. There are technical constraints, such as resolution and field of view issues. Current AR devices have a limited field of view and resolution [21-24] and, thus, do not yet provide the high precision and extensive field of view demanded in the medical field. Furthermore, although accurate position tracking and superimposition are essential in medical practice, errors in surgery at sites of variable positions currently limit their application. Another challenge is compatibility issues with existing systems. Integration with existing medical information systems and devices involves technical challenges.

The development described in the present study may be a solution for intraoperative positional corrections. The modification of the tracking system significantly improved tracking accuracy. Many of the methods reported in previous studies were for areas such as the spine and pelvis, where intraoperative movement is minimal. Few studies described adapting AR technology for the upper extremities because of the difficulties associated with the tracking of moving parts, which results in failure. This method, which uses a 3D camera with real-time intraoperative information updates, may be a solution to the challenges of AR on moving parts of the body, such as the upper extremities. The results obtained showed that the average errors of the reference points were <2.0 mm in the modified system. This accuracy is considered to be at a level close to clinical applications. Another advantage of this technology is that it enables tracking based on markers attached to the body surface. This eliminates the need for invasive markers, such as pins that are fixed to bone, as used in previous studies [25-27]. In addition, the system developed herein is designed to operate on AR glasses by setting a 3D camera in the surgical field, offering convenience because it does not require a large-scale system.

The 3D sensor used in the present study was a stereo vision 3D sensor. This method measures the distance from the parallax of two image sensors. The stereo vision method was selected from various 3D sensors because it is capable of real-time processing and is resistant to environmental noise. In the present study, a diffractive optical element laser was used to improve the performance of the stereo vision 3D camera by emitting textures (random dots and other patterns) with an infrared laser. This resolves the disadvantage of stereo vision, which is the inability to measure the distance of untextured objects. While other 3D sensor systems require graphics processing unit and personal computer resources for post-processing, this sensor features 3D data directly from the sensor. These are the advantages of stereo vision 3D sensors. An increased processing speed and higher-resolution 3D depth information have improved tracking accuracy. This brings the clinical application of AR vision to real-time update imaging one step closer.

Limitations

There are still several limitations that need to be addressed for the clinical application of the technology developed in this study. There was a slight temporal mismatch in corrections for position changes. While it is possible to track changes in a surgical site, there is a one- to two-second delay. This issue necessitates an increase in the analysis speed of the program. Another disadvantage is that the sharpness of visualization decreases depending on the distance to the object. Furthermore, challenges are associated with position corrections for soft tissue displacement. Further studies are needed on how to compensate for changes in reference points on the body surface due to incisions or the treatment of surgical sites. The current approach is to consider reference points in areas where tissue displacement is less likely; however, other methods need to be examined. Moreover, for future functional expansion, the plan is not only to use the field of view of a 3D camera as the surgical field but also to develop a system that uses AR glasses (with built-in cameras) as the surgical field, projecting preoperative 3D images in real time. This will allow these images to be overlaid on AR glasses without the need to move the gaze from the surgical field to a monitor screen.

## Conclusions

AR technology using a 3D camera has been developed. This system allows direct comparisons of 3D data from preoperative CT scans with 3D data acquired from the surgical field using a 3D camera. The modified system was able to estimate the position of the surgical field with an average error of 1.4 mm, corresponding to changes in the position of the surgical field. However, there are still several challenges in integrating 3D cameras into AR systems. These include high computational demands, sensitivity to lighting conditions, concerns about accuracy, and issues related to effective deployment and adaptation. This method represents an advancement in the potential to introduce the latest AR imaging to the surgical field without using invasive reference markers.
